# Feels Like Home: A Biobased and Biodegradable Plastic Offers a Novel Habitat for Diverse Plant Pathogenic Fungi in Temperate Forest Ecosystems

**DOI:** 10.1007/s00248-024-02466-0

**Published:** 2024-12-21

**Authors:** Paradha Nonthijun, Benjawan Tanunchai, Simon Andreas Schroeter, Sara Fareed Mohamed Wahdan, Eliane Gomes Alves, Ines Hilke, François Buscot, Ernst-Detlef Schulze, Terd Disayathanoowat, Witoon Purahong, Matthias Noll

**Affiliations:** 1 Institute of Bioanalysis, Coburg University of Applied Sciences and Arts, Coburg, Germany; 2 Department of Biology, Faculty of Science, Chiang Mai University, Chiang Mai, 50200 Thailand; 3 Max Planck Institute for Biogeochemistry, Biogeochemical Processes Department, Hans-Knöll-Str. 10, 07745 Jena, Germany; 4 Department of Botany and Microbiology, Faculty of Science, Suez Canal University, Ismailia, 41522 Egypt; 5 Department of Soil Ecology, UFZ-Helmholtz Centre for Environmental Research, Theodor-Lieser-Str. 4, 06120 Halle (Saale), Germany; 6 German Centre for Integrative Biodiversity Research (iDiv) Halle-Jena-Leipzig, Deutscher Platz 5E, 04103 Leipzig, Germany; 7 Center of Excellence in Microbial Diversity and Sustainable Utilization, Faculty of Science, Chiang Mai University, Chiang Mai, Thailand; 8 Research Center of Deep Technology in Beekeeping and Bee Products for Sustainable Development Goal (SMART Bee SDGs), Chiang Mai University, Chiang Mai, Thailand

**Keywords:** Biodegradable plastic, Fungal pathobiomes, Next-generation sequencing, Forest ecosystems

## Abstract

Poly(butylene succinate-co-adipate) (PBSA), a biodegradable plastic, is significantly colonized and degraded by soil microbes under natural field conditions, especially by fungal plant pathogens, raising concerns about potential economic losses. This study hypothesizes that the degradation of biodegradable plastics may increase the presence and abundance of plant pathogens by serving as an additional carbon source, ultimately posing a risk to forest ecosystems. We investigated (i) fungal plant pathogens during the exposure of PBSA in European broadleaved and coniferous forests (two forest types), with a specific focus on potential risk to tree health, and (ii) the response of such fungi to environmental factors, including tree species, soil pH, nutrient availability, moisture content, and the physicochemical properties of leaf litter layer. Next-generation sequencing (NGS) revealed that PBSA harbored a total of 318 fungal plant pathogenic amplicon sequence variants (ASVs) belonging to 108 genera. Among the identified genera (*Alternaria*, *Nectria*, *Phoma*, *Lophodermium*, and *Phacidium*), some species have been reported as causative agents of tree diseases. *Plenodomus* was present in high relative abundances on PBSA, which have not previously been associated with disease in broadleaved and coniferous forests. Furthermore, the highest number of fungal plant pathogens were detected at 200 days of PBSA exposure (112 and 99 fungal plant pathogenic ASV on PBSA degraded under *Q*. *robur* and *F*. *sylvatic*-dominated forest, respectively), which was double compared mature leaves and needles from the same forest sites. These findings suggest that PBSA attracts fungal plant pathogens in forests as an additional carbon source, potentially leading to increased disease outbreaks and disrupting the stability of forest ecosystems. The fungal plant pathogenic community compositions were mainly shaped by forest type, PBSA exposure time, site locations, leaf litter layer water content, and N:P ratio from leaf litter layer in both forest types. This study provides valuable insights into the potential risks posed by biodegradable plastic degradation in forests after 200 and 400 days of exposure, respectively. Further comprehensive evaluations of their effects on tree health and ecosystems, ideally on a long-term basis, are needed. These evaluations should include integrating microbial investigation, soil health monitoring, and ecosystem interaction assessments. Nevertheless, it should be noted that our interpretation of plant pathogens is solely based on high-throughput sequencing, bioinformatics, and annotation tools.

**Supplementary Information:**

The online version contains supplementary material available at 10.1007/s00248-024-02466-0.

## Introduction

The escalating demand for consumer items and industrial operations has led to a surge in the manufacturing and application of plastics, resulting a widespread environmental presence in both terrestrial and aquatic environments after use [1]. Forests encompass over 38% of the Earth’s land surface and are a crucial component of terrestrial ecosystems [2]. In addition to timber production, they have an impactful ecological meaning by harboring a huge biodiversity, functioning as both a source and sink of carbon (C), regulating rainfall, and exchanging atmospheric gases [3]. Therefore, appropriate management of forests is essential for both the economic and environmental conservation sectors. The distribution of plastics in forests has become a serious environmental issue, driven by human activities and inadequate waste management practices [4, 5]. Due to their resistance to degradation, plastics persist in the environment, leading to significant pollution [6]. Plastic pollution in agricultural soils has been extensively studied, with evidence showing alterations in soil physicochemical properties [7], and shifts in soil microbial communities, potentially impacting crop yields [8, 9]. Nevertheless, other forms of soil pollution, such as heavy metal contamination, pesticide residues, and nitrogen deposition, have been well-documented to influence soil microbial communities negatively impacting nutrient cycling and ecosystem health [10, 11]. Similar concerns have been raised regarding aquatic ecosystems, where plastics contribute to water pollution and threaten biodiversity [12]; however, the impacts of plastic pollution in forest ecosystems remain less examined. To address this issue, the development of biodegradable plastics derived from natural sources has gained attention in the past few years.

Biodegradable plastics can be degraded in natural soils through microbial activities from bacteria and fungi [13]. Biodegradable plastics, derived from both renewable and non-renewable sources, have evolved as alternatives to conventional plastics because of their degradability under different environmental conditions [14]. The most significant group of biodegradable polymers currently used is the family of aliphatic polyesters, including poly(hydroxyalkanoates) (PHAs), poly(L-lactic acid) (PLA), poly(butylene succinate) (PBS), and poly(butylene adipate) (PBA) [15]. Aliphatic polyester PBS and its well-known copolymer poly(butylene succinate-co-adipate) (PBSA) are among the most promising candidates for degradation in the environment [16]. Over the past decade, PBS and PBSA have been produced from petrochemical sources; nowadays, they tend to be produced from renewable resources such as sugarcane, cassava, and corn as bio-based alternatives [17]. It is suitable for a wide range of applications because of its similar properties to petroleum-based low-density polyethylene [18]. For example, they are used to produce films for product packaging and mulch films [19]. PBSA has been used in the forest industry to produce nursery containers or plant pots used for cultivation of tree seedlings before their transplanting into forest habitats [20]. As these containers are employed for nurturing tree seedlings, they often end up discarded or left behind after transplantation in forests. This can result in the accumulation of plastic in forested areas, thereby contributing to plastic pollution. The effect of plastic pollution is controversial as some studies have revealed that biodegradable plastics may not have harmful effects on the environment [21, 22]. However, other studies have shown that biodegradable plastics can produce microplastics, which can have negatively effects on microbial diversity and plant health [23, 24].

Microorganisms, especially fungi, are involved in the biodegradation of plastics because they can attach to and colonize the plastic surface [25] and degrade it by secreting strong extracellular enzymes that can penetrate and break down the plastic polymers [26–28]. Such enzymes include cutinases, lipases, and esterases [29], which can degrade plastics into smaller polymer intermediates and convert them into oligomers, monomers, water, and carbon dioxide (CO_2_) [30, 31]. Plastic degradation in soil provides additional carbon resources, creating favorable conditions for fungi that utilize these resources, promoting their persistence on plastics over time [16, 28, 32]. Additionally, Purahong et al. [8] suggest that PBSA can support distinct microbial communities, potentially affecting nutrient cycling and soil health. As these communities adapt to use PBSA as a nutrient source, shifts in biodiversity may occur, favoring species that degrade these materials and potentially disadvantaging native species. Known fungal species with the potential to degrade plastics include *Aspergillus niger*, *A. flavus*, *A. oryzae* [33], *A. fumigatus*, *Fusarium solani*, *Pleurotus ostreatus*, *Penicillium chrysogenum* [34], and *P. griseofulvum* [29]. Interestingly, most of the fungi reported to be involved in plastic degradation also belong to the group of plant pathogenic species [35–37]. Plant pathogens have often been reported to be capable of degrading plastics [38, 39]. In agricultural field conditions, Juncheed et al. [9] investigated bacteria and fungi associated with PBSA degradation to identify and evaluate the presence of microbial plant pathogens. The important fungal plant pathogens, they detected in PBSA were *Alternaria alternata*, *A*. *hordeicola*, *Cladosporium herbarum*, *Clonostachys rosea*, and *F*. *solani*. The observation of fungal plant pathogens in both plastic-amended and non-plastic-amended soils indicates their natural presence in the environment. Moreover, biodegradable plastics might unintentionally support the persistence of these pathogens, potentially threatening crop health and agricultural productivity [9]. In contrast, the impact of fungal pathogens associated with PBSA degradation in forest ecosystems has not yet been thoroughly examined. However, fungi such as *A. fumigatus* [30], *A. oryzae* [40], *Cladosporium* [16], *F. solani* [41], and *Penicillium* [42] have been commonly reported on PBSA environments. Fungal plant pathogens play a significant role in the health and well-being of forest ecosystems by influencing the fitness and function of their plant hosts [43] and by affecting the host’s C uptake and biomass production [42]. The impact of plant pathogens in forests extends beyond ecological concerns and encompasses significant economic repercussions [44]. In addition, they have led to increased costs associated with disease management and control measures [45]. However, the effects of plant pathogens associated with plastic degradation in forest ecosystems have rarely been explored. The specialized natural enemies (such as pathogens or herbivores) target specific plant species or individuals in species-rich communities (the Janzen-Connell hypothesis) [46, 47]. These natural enemies can reduce the survival and growth rate of seeds and seedlings in areas near conspecific adults or in areas of high conspecific tree density, thereby maintaining the diversity of plant communities in forests [48, 49]. However, in anthropogenically managed forests where tree biodiversity is low, the influence of pathogens may be increasingly significant for tree biodiversity loss [50]. Hence, comprehending the composition and presence of plant pathogens in plastic is crucial for assessing potential risks to plant health, further evaluating the environmental risks associated with plastic pollution, and implementing knowledge-driven campaigns of plastic reduction in forest ecosystems. In this investigation, we focus on temperate-managed forests, which are subjected to lower tree diversity as compared to unmanaged forests.

Environmental factors, including soil pH, water content, and temperature, play a significant role in shaping the microbiome of the plastisphere on bio-based and biodegradable plastics that degrade in agricultural soils [8, 51]. In forest ecosystems, Goldmann et al. [52] reported that the difference in dominant tree species and soil properties significantly affects the soil microbial community, which may potentially influence the presence of microorganisms that can colonize plastics. Tanunchai et al. [16] also indicated that forest types and decomposition time of plastics in forests affect the microbial community composition of the plastisphere microbiome in forest ecosystems.

This study fills important research gaps in understanding the role of biodegradable plastic, specifically PBSA, in enhancing fungal plant pathogens in forest ecosystems. Prior research focused on the biodegradation of plastics in agricultural and aquatic environments, but limited attention has been given to forest ecosystems. We initially addressed this gap by investigating how PBSA degradation impacts fungal plant pathogen communities in both broadleaved and coniferous forest sites, and how environmental factors such as tree species, soil pH, and nutrient availability shape these communities. In this study, we re-analyzed a published dataset on fungal communities involved in PBSA degradation [16], focusing specifically on fungal plant pathogens. The published dataset examined PBSA films placed on the leaf litter in forest sites dominated by broadleaved species (*Fagus sylvatica* (pBU) and *Quercus robur* (pEI)) and coniferous species (*Picea abies* (pFI) and *Pinus sylvestris* (pKI)). We aimed to understand how environmental factors, such as tree species, soil pH, nutrient availability, moisture content, and the physicochemical properties of the leaf litter, influence the composition and abundance of fungal plant pathogens associated with PBSA degradation.

## Materials and Methods

### Study Site, Experimental Setup, Designs, and Environmental Parameters

Details of the experimental setup have been published by Tanunchai et al. [51]. Briefly, the study site was in a managed mixed forest in Thuringia, Germany (51° 12′' N, 10° 18′ E), with mean annual precipitation ranging from 600 to 800 mm, mean annual temperatures ranging from 6 to 7.5 °C, and elevations from 100 to 494 m above sea level. The soil pH was acidic (5.1 ± 1.1; mean ± SD). PTT MCC Biochem Company Limited, Thailand, provided PBSA films (BioPBS FD92) as double-layer thin films with a thickness of 50 μm and 35% bio-based C (from corn). In November 2019, PBSA films were placed on the leaf litter layer under the canopy of four different trees in a forest dominated by beech (*Fagus sylvatica*, BU), oak (*Quercus robur* (EI), spruce (*Picea abies*, FI), and pine (*Pinus sylvestris*, KI) with five independent biological replicates for each tree type. *F. sylvatica*, *Q. robur*, *P. abies*, and *P. sylvestris* were selected in this pioneering study as they represent the most widespread species in global and European temperate forests [53, 54], and are of significant value to forest productivity, ecology, and economy [55]. After 200 and 400 days of PBSA exposure at each forest site, PBSA films and soil samples were collected in separate sterile plastic bags, transported on ice to the laboratory within 3 h, and stored at − 80 °C for further analyses. The soil pH and water content were determined and used as explanatory variables for microbial responses. More details on the study site, experimental setup, design, and environmental parameters are provided in the Supplementary Material.

### Characterization of the Fungal Plant Pathogenic Plastisphere Microbiome

The Illumina-based sequencing approach for plastisphere microbiome analysis has been previously published [8, 51]. The Qiagen DNA extraction kit showed a high yield of DNA concentrations at relatively low costs and short processing time [56]. In this study, we used the same established DNA extraction method to compare the plastisphere across different habitats. The extraction of soil microbiome is out of the scope of this study due to a limited budget and should be extended in the future study. Briefly, the PBSA samples were randomly cut in 12.5-cm^2^ pieces and cleaned. The DNeasy PowerSoil Kit (Qiagen, Hilden, Germany) was used to extract DNA from the microbial biomass that was firmly attached to the PBSA sample following the manufacturer’s instructions. The presence and quantity of genomic DNA was checked using the NanoDrop ND-1000 spectrophotometer (Thermo Fisher Scientific, Dreieich, Germany). For fungal amplicon library preparation, the internal transcribed spacer 2 (ITS2) region was amplified using the fungal universal primer pair fITS7 (5′-GTGARTCATCGAATCTTTG-3′) [57] and ITS4 (5′-TCCTCCGCTTATTGATATGC-3′) [58] with Illumina adapter sequences. Amplifications were performed in 20-μL reactions with 5 × HOT FIRE Pol Blend Master Mix (Solis BioDyne, Tartu, Estonia). The amplified products were visualized by gel electrophoresis and purified using an Agencourt AMPure XP kit (Beckman Coulter, Krefeld, Germany). Paired-end sequencing of 2 × 300 nucleotides from this pool (three technical replicates) was performed using a MiSeq Reagent kit v3 on an Illumina MiSeq system (Illumina Inc., San Diego, CA, USA) at the Department of Soil Ecology, Helmholtz Centre for Environmental Research. The ITS rRNA gene sequences were subjected to bioinformatics analysis. More details on bioinformatics are provided in the Supplementary Material. Briefly, singletons or sequences that potentially represented artificial sequences were removed from the dataset. After rarefication, we obtained 2,683 rarefied fungal amplicon sequence variants (ASVs). Rarefaction curves demonstrated an adequate sequencing depth level for all samples (Figure S1). Thus, we used the observed richness as a measure of fungal plant pathogen diversity associated with PBSA degradation. Ecological functions of each ASV were determined using FungalTraits [59, 60]. Fungal plant pathogens were separated from other functional groups and used in this study.

### Physiochemical Analyses of Leaf Litter Layer

As PBSA is degraded in the leaf litter layer, we measure the leaf litter layer nutrients (C, N, Ca, Fe, K, Mg, and P) to investigate the effect of environmental factors on PBSA. The plastic degradation experiment was conducted in the leaf litter layer rather than in the soil because, in real-world scenarios, plastic waste entering a forest accumulates on the topsoil, which is primarily covered by the litter layer—where microbial activity is highest on the forest floor. The methods used for leaf litter layer physicochemical analyses have been published in previous study [61]. To prepare for measuring Ca, Fe, K, Mg, and P, 100 mg of sample material was subjected to a microwave-assisted high-pressure digestion (Multiwave 3000, Anton Paar, Graz, Austria) at a maximum microwave power of 1200 W and a maximum pressure of 60 bar following the addition of 3–5 mL 65% HNO_3_, supra-pur (Merck, Darmstadt, Germany). Rotor 8SXF100 with reaction vessels made of TFM (tetrafluor-modified polytetrafluoroethylene) was used. The total digesting time was 50 min, including 20 min of cooling at a microwave power of zero. To check for reagent and vessel contamination, a blank of solely nitric acid was used. Following digestion, the solutions were filtered and transferred to 50-mL PE containers filled to the mark with ultrapure water (Millipore, Eschborn, Germany). The sample solution analyses were then performed using inductively coupled plasma–optical emission spectrometry (ICP-OES) “Arcos” (Spectro, Kleve, Germany) equipped with a 27.12 MHz free-running LDMOS generator and ORCA optical system. A three-point calibration was performed using single-element standards given by Merck (Darmstadt, Germany) at the following concentrations: 10, 50, and 100 mg/L for Ca, K, Mg, and P, and 0.5, 2.5, and 5 mg/L for Fe. The total C and N contents were determined using an Elementar Vario EL III (Elementar Analysensysteme GmbH, Langenselbold, Germany). The total C and N contents were analyzed using an Elementar Vario EL III (Elementar Analysensysteme GmbH, Langenselbold, Germany).

### Statistical Analysis

The effects of forest type and exposure time on fungal plant pathogenic relative abundance and fungal plant pathogenic ASVs richness were assessed through repeated analysis of variance (ANOVA) analysis using SPSS (as the dataset varied by time series), incorporating the Jarque–Bera JB test for normality and Levene’s test to assess the equality of group variances. The effects of soil and litter layer physiochemical properties on fungal plant pathogenic community compositions on PBSA samples were visualized using non-metric multidimensional scaling (NMDS) and tested using goodness-of-fit statistics based on observed relative abundance data and the Bray–Curtis distance measure.

## Results

### Fungal Plant Pathogens Detected in PBSA After 200 and 400 Days of Exposure

**Fig. 1:**
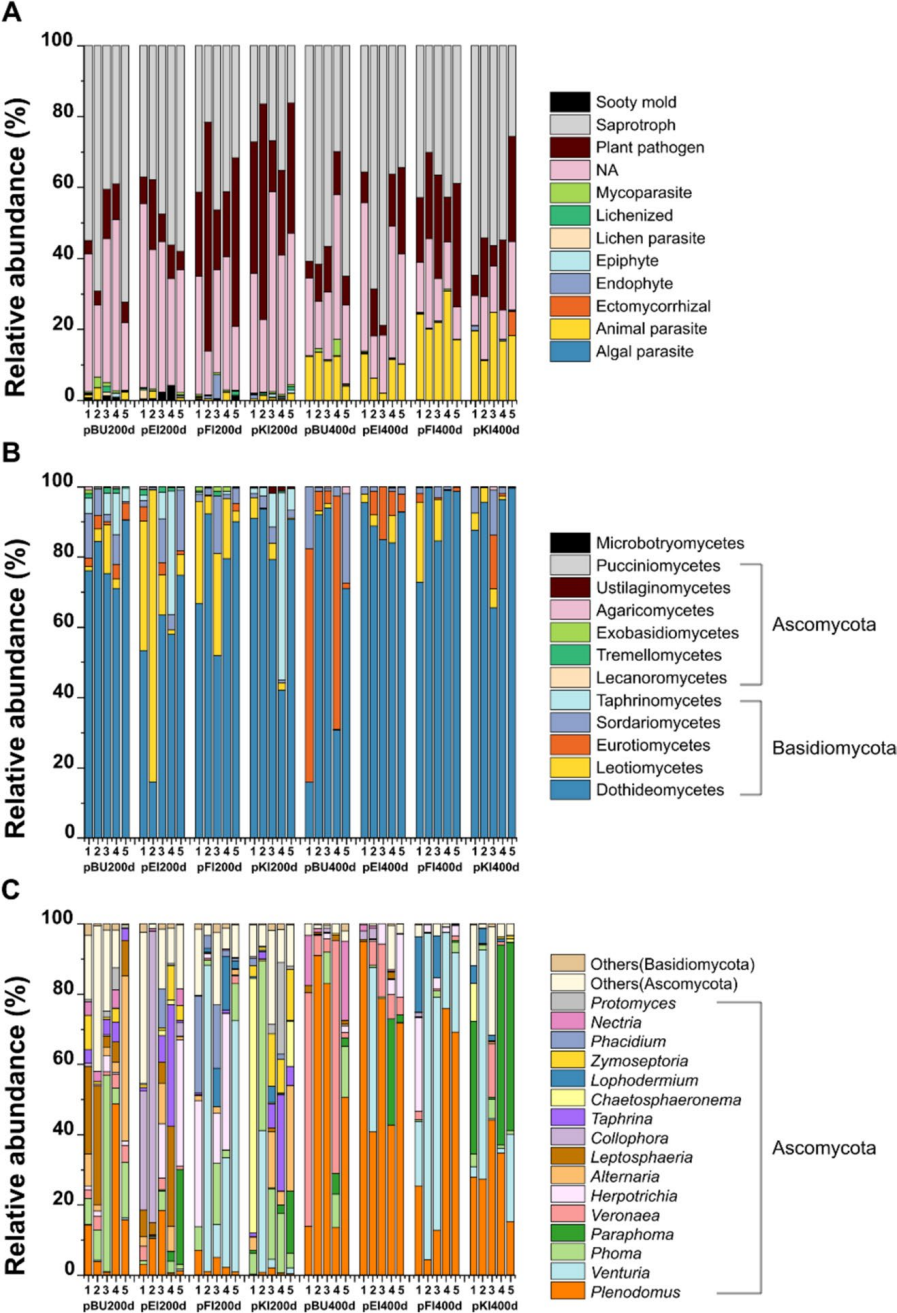
Composition of overall fungal functional groups (**A**) and composition of fungal plant pathogens [class level (**B**) and genus level (**C**), considering only classes or genera with ASV relative abundances ≥ 1%; the rest of the fungal classes and genera were pooled as “others”] on poly(butylene succinate-co-adipate) (PBSA) after 200 and 400 days of exposure under four tree species *Fagus sylvatica* (pBU), *Quercus robur* (pEI), *Picea abies* (pFI), and *Pinus sylvestris* (pKI) at two sampling times (200 and 400 days). Functional annotation was carried by using FungalTraits

Apart from fungal saprotrophs, fungal plant pathogens contributed to the second highest relative ASV abundance, especially in coniferous trees at 200 days (Fig. 1A). Overall, PBSA plastics under different tree species at 200 and 400 days were colonized by 2,683 fungal ASVs, of which 318 were classified as fungal plant pathogens. The 318 ASVs belonged to 108 genera (Table S1). The fungal plant pathogenic community detected in PBSA was dominated by Dothideomycetes (represented by *Plenodomus*, *Venturia*, *Phoma*, and *Paraphoma*). These groups contributed up to 77% of the total fungal plant pathogenic relative abundance across the different forest types (broadleaved or coniferous forests) and exposure times (Fig. 1B, Fig. 4, and Table S2). Interestingly, Leotiomycetes (represented by *Collophora*, *Lophodermium*, and *Phacidium*) were highly detected on PBSA degraded under *Q*.* robur* dominated forest (pEI) at 200 days but were almost absent after 400 days of exposure. In contrast, Eurotiomycetes, represented by *Veronaea*, were highly detected in *F*.* sylvatica* dominated forest (pBU) at 400 days (Fig. 1C, Fig. 4, and Table S2). Our results revealed three distinct patterns of fungal plant pathogens. First, the relative abundance of *Plenodomus* was low at 200 days but increased (over 25%) at 400 days across the different forest types (Fig. 4 and Table S3). Second, the fungal genera *Alternaria*, *Leptosphaeria*, *Collophora*, *Taphrina*, *Chaetosphaeronema*, and *Zymoseptoria* were detected at 200 days, but they almost completely absent at 400 days. Finally, in coniferous forest type, the relative abundances of *Venturia*, *Lophodermium*, and *Phacidium* were observed across PBSA exposure times (Fig. 1C and Fig. 4).

### Distinct Fungal Plant Pathogens Have Been Associated with PBSA Degradation in Temperate Forests

**Fig. 2:**
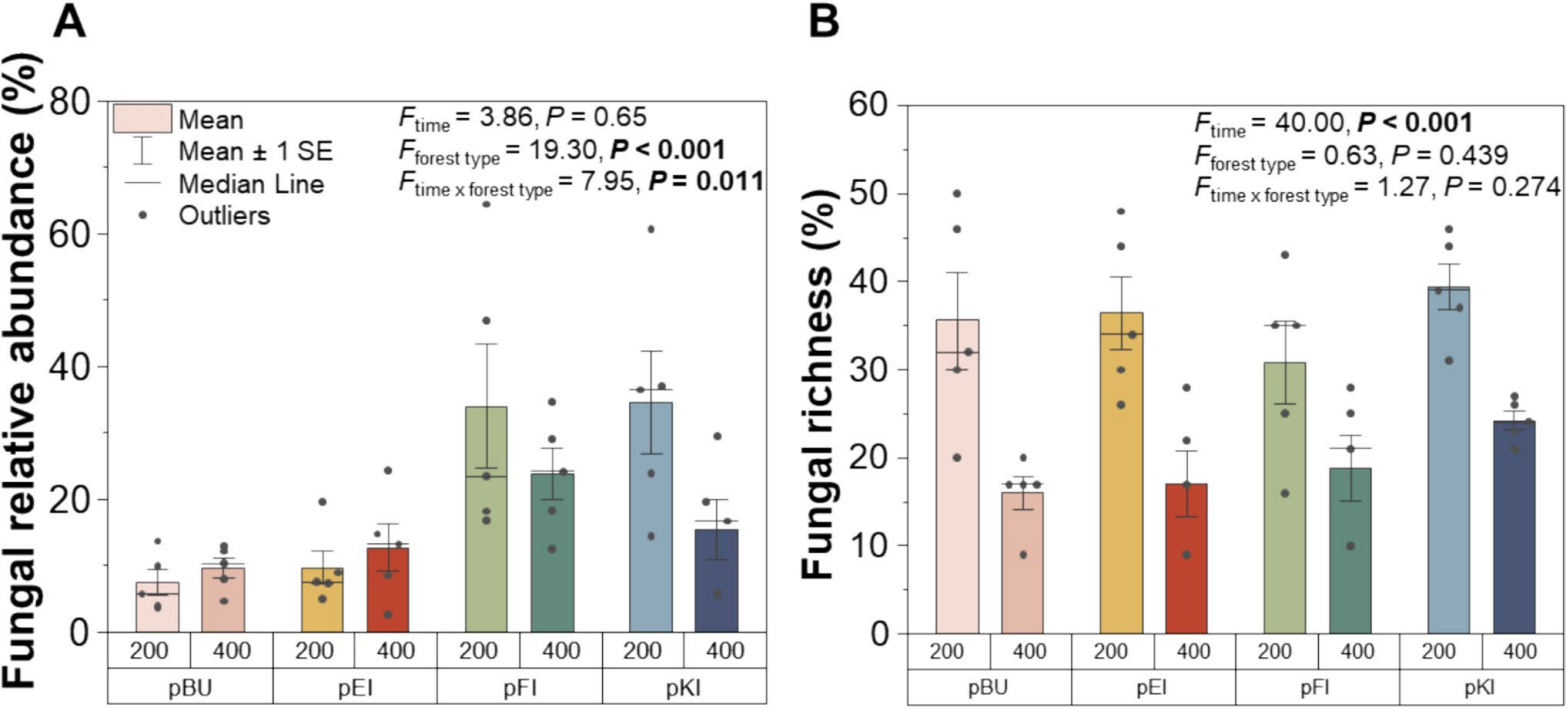
The fungal plant pathogenic ASV relative abundance (**A**) and fungal plant pathogenic ASV richness (**B**) on PBSA after 200 and 400 days of exposure under four tree species *Fagus sylvatica* (pBU), *Quercus robur* (pEI), *Picea abies* (pFI), and *Pinus sylvestris* (pKI) at two sampling times (200 and 400 days). Standard error and median line of five replicate measurements are indicated

At 200 days of PBSA exposure, the fungal plant pathogenic relative ASV abundance in coniferous forest type was higher (34.0 ± 9.4 to 34.5 ± 7.8; mean ± SE) compared to broadleaved forest type (7.4 ± 1.9 to 9.7 ± 2.6; mean ± SE; Fig. 2A). In all forest sites, higher plant pathogenic ASV richness on PBSA were detected at 200 days (30.8 ± 4.7 to 39.4 ± 2.7; mean ± SE), which significantly decreased after 400 days of exposure (16.0 ± 1.8 to 24.2 ± 1.1; mean ± SE, Fig. 2B).

### Forest Type Affect Community Composition of Fungal Plant Pathogens

**Fig. 3:**
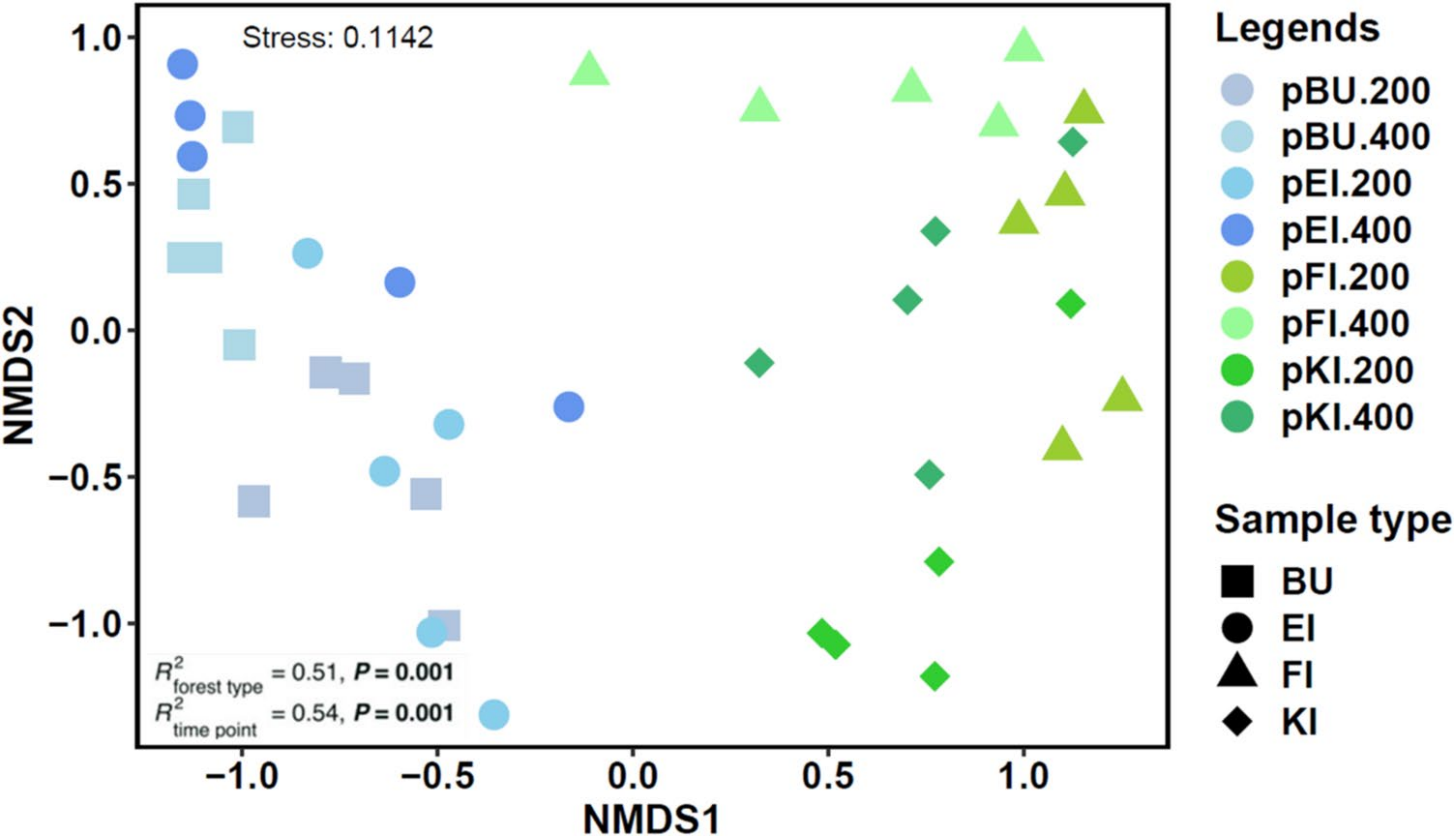
Non-metric multidimensional scaling (NMDS) ordinations of fungal plant pathogenic community composition in poly(butylene succinate-coadipate) (PBSA) under all tree species, based on relative sequence abundance data and Bray–Curtis distance measures. Blue tones: broadleaved trees; green tones: coniferous trees. Data are presented for PBSA samples under four tree species *Fagus sylvatica* (pBU), *Quercus robur* (pEI), *Picea abies* (pFI), and *Pinus sylvestris* (pKI)

Forest type (*R*^2^ = 0.51, *P* = 0.001) and exposure time (*R*^2^ = 0.54, *P* = 0.001, Table S4) significantly affected the fungal plant pathogenic community composition. NMDS based on Bray–Curtis similarity was used to compare the fungal plant pathogenic community composition on PBSA plastic after 200 and 400 days of exposure. Fungal plant pathogens present in broadleaved and coniferous forest types exhibited notable differences in community composition (Fig. 3).

### PBSA as Home for Fungal Plant Pathogens in Temperate Forests

We observed a notable presence of fungal plant pathogens in PBSA degraded in the forest. At 200 days of PBSA exposure, *Q. robur*-dominated forest harbored the highest number of fungal plant pathogenic ASVs (112 ASVs), followed by *F. sylvatica*-dominated forest (99 ASVs). *P. abies* and *P. sylvestris*-dominated forest showed an equal number of fungal plant pathogenic ASVs (83 ASVs). The fungal plant pathogenic ASVs at 400 days of each tree species significantly decreased. *Q. robur* and *P. sylvestris*-dominated forest harbored 61 ASVs of fungal plant pathogens, followed by *P. abies* (48 ASVs) and *F. sylvatica* (44 ASVs). Among the 318 fungal plant pathogenic ASVs, 216 ASVs were connected to only one tree species, indicating the specialist nature. While 11 ASVs were detected in all four tree species, demonstrating a generalist capability (Figure S2 and Table S5).

### Factors Determining Fungal Plant Pathogenic Community Composition

The exposure time, site locations, leaf litter layer water content, and N:P ratio significantly affected the fungal plant pathogenic community composition in both forest types (*R*^2^ = 0.38–0.74, *P* < 0.05–0.001, Table 1). The PBSA exposure time was the main factor shaping the composition of the fungal plant pathogen community in broadleaved forest type (*R*^2^ = 0.74, *P* < 0.001). The soil water content, leaf litter layer water content, leaf litter layer pH, Fe content, and N:P ratio were also significantly correlated with the fungal plant pathogenic community composition in broadleaved forest type (*R*^2^ = 0.40–0.63, *P* < 0.01–0.001, Table 1). The fungal plant pathogen community obtained in coniferous forest type was mainly shaped by latitude, longitude, and leaf litter layer water content (*R*^2^ = 0.70–0.74, *P* < 0.001, Table 1). Dominating tree species, tree type, timepoint, plot factors, and factors from the leaf litter layer (except N, Ca content, and C:N ratio) also significantly shaped the fungal plant pathogenic community in the plastisphere (*R*^2^ = 0.21–0.66, *P* < 0.05–0.001, Table S4).

**Table 1:** Goodness-of-fit statistics (*R*^2^) of environmental and PBSA variables fitted to the nonmetric multidimensional scaling (NMDS) ordination of fungal plant pathogenic community based on relative abundance data and the Bray–Curtis distance measure

Factors	Broadleaved forest type	Coniferous forest type
Plot factors		
Tree species	0.01	0.42***
Soil water content	0.51**	0.21
Soil pH	0.26	0.60***
Latitude	0.14	0.70***
Longitude	0.53**	0.70***
PBSA factor		
Exposure time	0.74***	0.53**
Leaf litter layer factors		
Leaf litter layer water content	0.40*	0.74***
Leaf litter layer pH	0.63***	0.30
C	0.11	0.41*
N	0.14	0.65**
Ca	0.01	0.39*
Fe	0.41**	0.12
K	0.07	0.53**
Mg	0.26	0.20
P	0.28	0.07
C:N ratio	0.21	0.64***
C:P ratio	0.25	0.10
N:P raio	0.45***	0.38*

Bold letters indicate statistical significance. *P < 0.05, **P < 0.01, and ***P < 0.001

## Discussion

### The Role of PBSA as a Novel Habitat for Fungal Plant Pathogens in the Forest Ecosystem

Presence of PBSA in forest ecosystems caused a temporal increase (Fig. 2) of fungal plant pathogens, which was significantly reduced over time. The decrease in fungal plant pathogen richness may be due to the alteration of polymer structures as degradation progresses and an overgrowth of some fungal plant pathogens, especially *Plenodomus*. This can lead to the emergence of various polymer-based and microbially induced metabolites, which in turn may favor the colonization of different fungi better adapted to these conditions, outcompeting the initial plant pathogens [8]. A previous study [62] also investigated the fungal community composition in leaves and needles at the same study site. They detected 52, 51, and 50 fungal plant pathogenic ASVs in leaves of *F. sylvatica*, and needles of *P. sylvestris*, and *P. abies*, respectively. Different patterns of fungal plant pathogen colonization between leaves and on PBSA may be attributed to the chemical compositions of the substrates. Leaves are primarily composed by cellulose, hemicellulose, lignin, sugars, fatty acids, organic acids, and mineral substances [63, 64]. These complex matrices provide a diverse range of C and energy sources for microbial communities. In contrast, plastics like PBSA are synthetic polymers made up of repeating units of the same monomers including, succinic acid, adipic acid, and 1,4-butanediol, which are derived from renewable resources such as corn and sugarcane [17, 65]. The distinct nature of the two substrate types could influence the distribution and activity of fungal communities. Importantly, PBSA-degrading fungi outcompete other fungal species in utilizing this synthetic substrate due to their capability to produce plastic-degrading enzymes [66, 67], providing them with a competitive advantage in colonizing and degrading plastic materials in forest environments. Although PBSA itself does not act as a hub for fungal plant pathogens, its degradation in forest ecosystems correlates with an increase in the number of fungal plant pathogens. We found the enrichment of fungal plant pathogens at 200 days of PBSA exposure. This period is the stage of decomposition that microbial activities for plastic depolymerization and the utilization of polymer C potentially occur [8]. These processes involve the breakdown of the long polymer chains into monomers, water, and CO_2_, which can be utilized by microbes [31, 68]. Furthermore, the decomposition of added organic materials, including bio-based and biodegradable plastics, in soil also stimulates the soil organic matter breakdown, called the “priming effect,” which results in a significant release of carbon from both the added plastic and the native soil organic matter [69]. Thus, it may be inferred that PBSA indirectly contributes to the proliferation of fungal plant pathogens by serving both as habitat and additional C source for fungal plant pathogenic inoculation in temperate forest ecosystems.

### Fungal Plant Pathogens Detected on PBSA During Degradation in Temperate Forests

**Fig. 4:**
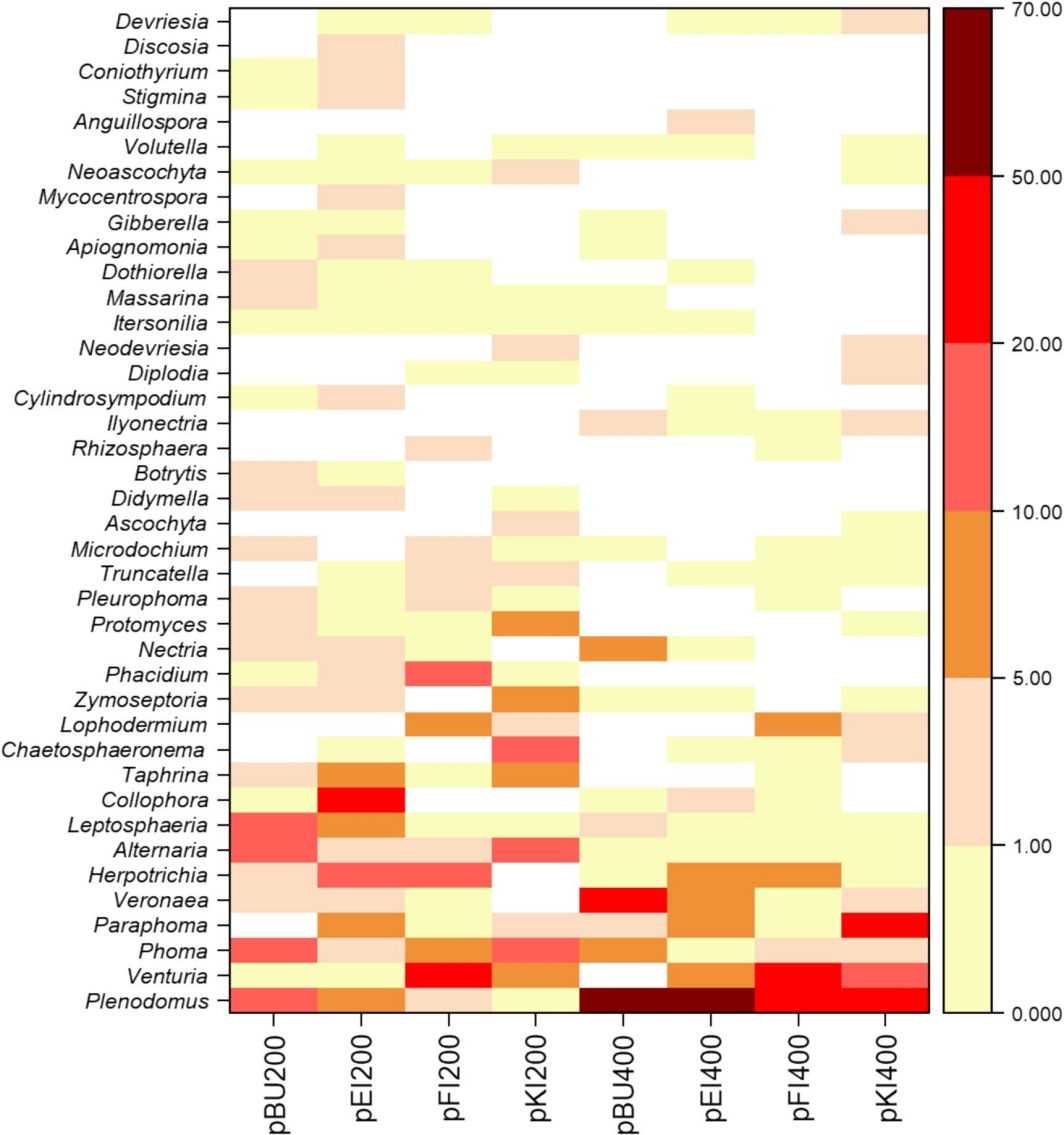
The occurrence of top 40 fungal plant pathogens detected on PBSA after 200 and 400 days of exposure under four tree species *Fagus sylvatica* (pBU), *Quercus robur* (pEI), *Picea abies* (pFI), and *Pinus sylvestris* (pKI). The yellow-red colour legend represent the respective relative sequence read abundances of each plant pathogen

This study provides insights into PBSA colonizers/decomposers that can act also as fungal plant pathogens. In line with previous studies, we detected dominant fungal plant pathogens belonging to genera *Plenodomus*, *Venturia*, *Phoma*, *Paraphoma*, *Veronaea*, *Herpotrichai*, and *Alternaria* on PBSA samples in our forest sites (Fig. 4). These fungi are often reported in the soil or on leaves/needles in undisturbed environments [62, 70–72]. Among the dominant fungal plant pathogen genera, *Plenodomus* showed the highest relative abundance at 400 days (45.9% of the fungal plant pathogens) across all tree species (Fig. 1C). This genus includes several well-known plant pathogens that affect economically significant crops, primarily in the Brassicaceae family [67], causing stem canker or blackleg in oilseed and wasabi crops (*P. lingam* and *P. biglobosus* [73]), and mal secco disease of twigs and branches in citrus plants (*P. tracheiphilus* [74, 75]). Although *Plenodomus* was abundant on PBSA during degradation in both forests, there are no reports of its pathogenicity in broadleaved and coniferous trees. *Phoma* has been reported as a fungal plant pathogen that causes root decay and stem and needle defoliation in *Pinus sylvestris*, *Pinus sibirica*, and *Picea obovata* [76]. Likewise, *Lophodermium* [77–80] and *Phacidium* [81–84] have also been reported as significant fungal plant pathogens in *P. sylvestris* and *P. abies*. Furthermore, *Alternaria* has been reported as a fungal plant pathogen in *F. sylvatica* [85] and *Q. robur* [86]. *Nectria* has been reported as the primary causal agents of root dieback in coniferous trees [87] and are associated with cambial necroses in *F. sylvatica* [88, 89]. Furthermore, some dominant fungal plant pathogens, such as *Plenodomus* [90], *Phoma* [91], and *Veronaea* [92] have demonstrated the ability to synthesize phytotoxins. These fungal plant pathogens, such as members of *Plenodomus* [93], *Venturia* [41, 94], *Phoma* [95, 96], *Paraphoma* [97, 98]*, Veronaea* [99], and *Alternaria* [14, 100] have been reported to produce lipase, cutinase, and/or esterase enzymes that are described to be effective for PBSA degradation. These enzymes can play different roles, depending on the substrate type. They can facilitate the fungal plant pathogenic infection on host plants [101–103]. On the other hand, these enzymes also play a crucial role in plastic degradation [66, 67]. This suggests that the fungal plant pathogens detected in this study can potentially serve as candidates for PBSA degradation in forest ecosystems.

### Environmental Factors Shaping the Fungal Plant Pathogenic Community

The environmental factors, including pH, nutrient availability, and moisture content, are one of the determinants that affect microbial community structure [104]. Furthermore, tree species identity are influential factors contributing to variations in microbial communities within both the forest floor and soil [105, 106]. These factors can influence the distribution and abundance of microbial taxa, ultimately shaping the dynamic patterns of microbial communities in the plastisphere [16, 107]. Fungal plant pathogenic community composition on PBSA exposed in both forest types was correlated to many factors, including tree species, exposure time, plot factors, and leaf litter layer factors (mainly site location, water content, pH, and N:P ratio, Table 1 and Table S4), which contribute also to different fungal plant pathogenic community patterns (Figs. 3 and 4). This finding aligns with a previous study [108] who investigated the susceptibility of trees to disease and the effects of forest site, tree type, and latitude on fungal plant pathogen community composition. In addition, a decrease in soil pH may limit fungal growth and reproduction, leading to an alteration in the fungal community composition [109, 110]. Proper water content is crucial and therefore shapes the diversity and composition of fungal communities, primarily through microbial dispersal and impact on soil texture and availability of nutrients necessary for fungal growth [110, 111]. Veresoglou et al. and Lekberg et al. [112, 113] indicated that variations in the N:P ratio in soil can affect the community composition of fungal plant pathogens, as high N content supports their growth and tends to increase their disease severity [36]. Fungi require phosphorus for vital cellular components such as chromosomes and ribosomes, as well as for producing ATP, the energy currency of the cell [114]. Thus, the availability of phosphorus in the soil can affect the growth and metabolism of fungi, leading to changes in their community composition [115–117].

## Practical Implication and Outlook

Plastic pollution is one of the serious environmental pollutions in terrestrial ecosystems. PBSA is an alternative bioplastic to replace conventional non-biodegradable plastics such as PE and PP due to their similar properties. They can be widely applied in terrestrial ecosystems such as in agriculture or forestry. As biodegradable plastics can be broken down into CO_2_ and water by microbes, they are expected to be left decomposed in the field site to save time and labor. Previous studies [9, 55] have raised concerns on the potential enrichment of fungal plant pathogens during PBSA degradation in agricultural soils. We aimed to compare this finding with PBSA degradation in temperate forest ecosystems to properly manage plastic waste. We found in both agricultural and forest ecosystems a similar pattern of the dominant fungal taxa that colonized PBSA [8, 9, 16]. These colonizers include *Phoma*, *Plenodomus*, and *Venturia*. However, these fungal colonizers can also act as pathogens for specific hosts, highlighting the impact on ecosystem health. PBSA could serve as a temporary habitat for fungal pathogens. Nevertheless, it should be noted that our interpretation of plant pathogens is based solely on high-throughput sequencing, bioinformatics, and annotation tools. Future studies should also use cultivation-dependent methods, including phenotypic characterization, to support these findings. Furthermore, microclimates such as UV light, air temperature, and humidity can also affect the colonization of fungal plant pathogens on PBSA. A previous study indicate that tropical regions exhibit unique environmental conditions, including higher microbial activity and specific soil properties [118] that may accelerate the degradation rates of biodegradable plastics. Conversely, unmanaged forests might exhibit different degradation dynamics due to minimal human intervention, resulting in more stable or complex microbial communities that could influence plastic degradation differently [119]. Specifically, in a humid subtropical climate, BioAgri (PBAT blended with starch) was already degraded by ∼20% after 6 months, but not in a Mediterranean climate with lower mean temperatures. The different microclimates may lead to physical and chemical property changes, and thus directly and/or indirectly affect the colonization of potential fungal plastic degraders/fungal plant pathogens. Thus, our findings may not fully transfer to the degradation of other bioplastics in other climate zones. pH is also one of the influencing factors that predict the composition of different natural substrates such as deadwood [120, 121], leaf, and needles [60]. A previous study [16] also showed that pH in soil and litter layer affects the microbial community compositions, and thus the degradation of PBSA plastic in broadleaved- and coniferous-dominated forests. Nevertheless, our studies enlighten that PBSA can be degraded in both agricultural and forest soils in the subcontinental climate region of Central Germany and have a similar pattern of fungal plant pathogenic colonization, even though the two study sites are located more than 100 km away from each other. Although the ITS marker is widely used and often regarded as the universal barcode for fungi due to its high variability across species, it is not sufficient for classifying all fungal species. This limitation arises from (i) its inability to reliably resolve closely related species and (ii) the availability of ITS reference sequences, which cover less than 1% of the estimated 6 million extant fungal species [122, 123]. Nevertheless, within the ribosomal cistron regions, the internal transcribed spacer (ITS) region offers the highest probability of accurate identification across a broad range of fungi, exhibiting the most distinct barcode gap between inter- and intraspecific variation [124]. For more precise classification, especially at the species level, additional markers or multilocus approaches—such as LSU (large subunit rRNA), SSU (small subunit rRNA), and protein-coding genes like TEF1, RPB1, and RPB2—can be employed.

Overall, we suggest removing plastics from forests after use regardless of their biodegradability and properly decomposing the plastic waste at the decomposing site. The investigation of plant pathogen colonization on during degradation should be extended to cover other plastic types and additional microclimate conditions. Evaluating the prolonged exposure of PBSA and other biodegradable plastics in different forest types to understand their potential effects on tree health, soil health, and overall biodiversity. Expanding studies to other forest ecosystems, which may exhibit different microbial community dynamics and plastic degradation processes due to varying environmental conditions. Potential long-term impacts on ecosystem biodiversity and resilience should be deeply investigated in long-term studies.

## Conclusion

This study revealed a broad diversity of fungal plant pathogens were detected on PBSA across forest types. PBSA, serving as a degradable substrate led to an enrichment of fungal plant pathogens, particularly noticeable after 200 days of PBSA exposure. However, a significant decline in fungal plant pathogen richness occurred after 400 days, suggesting a temporary enrichment phenomenon. The fungal plant pathogenic community was primary influenced by soil and leaf/needle-based parameters, underscoring the critical role of environmental conditions in forest ecosystems, as observed in leaf and deadwood decomposition processes. These finding offer valuable insights for knowledge-based seminars aimed at improving forest cleaning strategies to combat plastic waste. Additionally, further investigation is warranted to determine whether these enriched fungal plant pathogens exert any adverse effects on the health of trees and shrubs, particularly in light of anticipated stress due to global warming.

## Supplementary Information

Below is the link to the electronic supplementary material.

Supplementary file1 (PDF 529 KB)

Supplementary file2 (XLSX 1680 KB)

## Data Availability

Fungal datasets from Illumina sequencing were deposited in The National Center for Biotechnology Information (NCBI) database under BioProject ID PRJNA890592.
