# Supplementary material for: Feels Like Home: A Biobased and Biodegradable Plastic Offers a Novel Habitat for Diverse Plant Pathogenic Fungi in Temperate Forest Ecosystems
Source: Microb Ecol. 2024 Dec 21;87:155. doi: 10.1007/s00248-024-02466-0 (PMC11663174; doi:10.1007/s00248-024-02466-0)
Supplement: Supplementary file 1 [file 248_2024_2466_MOESM1_ESM.pdf]

Supplementary material

Research article

Title: Feels like home: A biobased and biodegradable plastic offers a novel habitat for diverse plant pathogenic fungi in temperate forest ecosystems.

Paradha Nonthijun[1,2,¶], Benjawan Tanunchai[1,¶], Simon Andreas Schroeter[3], Sara Fareed Mohamed Wahdan[4], Eliane Gomes Alves[3], Ines Hilke[3], François Buscot[5,6], Ernst-Detlef Schulze[3], Terd Disayathanoowat[2,7,8*], Witoon Purahong[5*], Matthias Noll[1*],

[1]Institute of Bioanalysis, Coburg University of Applied Sciences and Arts, Coburg, Germany.

[2]Department of Biology, Faculty of Science, Chiang Mai University, Chiang Mai 50200, Thailand.

[3]Max Planck Institute for Biogeochemistry, Biogeochemical Processes Department, Hans-Knöll-Str. 10, 07745 Jena, Germany.

[4]Department of Botany and Microbiology, Faculty of Science, Suez Canal University, 41522 Ismailia, Egypt.

[5]Department of Soil Ecology, UFZ-Helmholtz Centre for Environmental Research, Theodor-Lieser-Str. 4, 06120 Halle (Saale), Germany.

[6]German Centre for Integrative Biodiversity Research (iDiv) Halle-Jena-Leipzig, Deutscher Platz 5e, 04103 Leipzig, Germany.

[7]Center of Excellence in Microbial Diversity and Sustainable Utilization, Faculty of Science, Chiang Mai University, Chiang Mai, 50200, Thailand.

[8]Research Center of Deep Technology in Beekeeping and Bee Products for Sustainable Development Goal (SMART Bee SDGs), Chiang Mai University, Chiang Mai, 50200, Thailand.

***Correspondence**: Terd.dis@cmu.acth; Witoon.purahong@ufz.de; Matthias.noll@hs-coburg.de, Tel.: 09561-317645

Material and methods

Study site, experimental setup, designs and environmental parameters

The study site is in a managed mixed forest of Thuringia, Germany (51°12'N 10°18'E) (see map below).

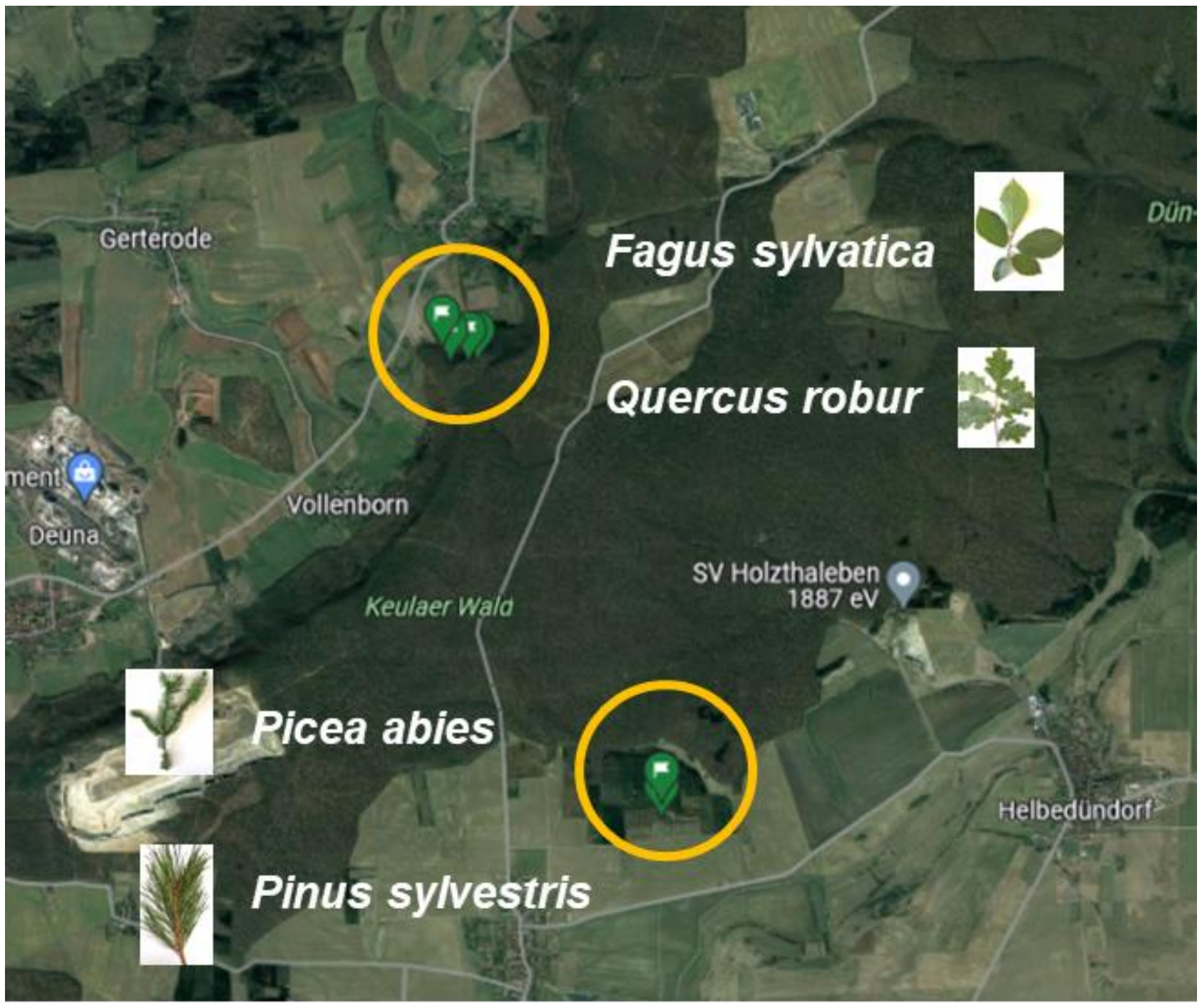

The location is characterized by mean annual precipitations from 600 to 800 mm, mean annual temperatures from 6 to 7.5°C, and elevations from 100 to 494 m above sea level. The main soil type is Cambisol on limestone as bed-rock. The soil pH is weakly acidic (5.1 ± 1.1; mean ± SD). In November 2019, the PBSA film (BioPBS FD92, PTT MCC Biochem Company Limited, Thailand; in the form of a double-layer thin film (21 cm × 30 cm) with 50 μm thickness; percent bio-based carbon, 35%) was placed on the top of the litter layer of four temperate forests each under the canopies of each tree (5 true replicates / tree individual per tree species). The four temperate tree species which were in the same study area in a managed mixed forest of Thuringia, Germany include two broadleaved tree species (BU: *Fagus sylvatica* and EI: *Quercus robur*) and two coniferous tree species (FI: *Picea abies* and KI: *Pinus sylvestris*). After 200 and 400 days of PBSA incubation in forest site, PBSA samples and soil samples under the PBSA films were collected in a separate sterile plastic bag. PBSA and soil samples were transported on ice to the laboratory within 3 h and stored at −80°C for further analysis. The soil pH and water content were determined and used as explanatory variables for microbial responses. Soil pH were measured in a 1:5 soil: water suspension using pH-meter (Hanna Instruments, Germany). Soil water content was determined by oven drying at 105°C for 48 h.

Characterization of the plastisphere

The individual technique for analyzing the plastisphere using Illunima Sequencing has been described elsewhere [1, 2]. Briefly, we randomly cut 12.5 cm^2 PBSA samples and cleaned the surface. Microbial biomass attached firmly to the PBSA sample was then subjected to DNA extraction using the DNeasy PowerSoil Kit (Qiagen, Hilden, Germany) with the aid of a Precellys 24 tissue homogenizer (Bertin Instruments, Montigny-le-Breton-neux, France). For the

fungal amplicon libraries, the fungal internal transcribed spacer 2 (ITS2) was amplified using the fungal primer pair fITS7 (5′-GTGARTCATCGAATCTTTG-3′) [3] and ITS4 (5′-TCCTCCGCTTATTGATATGC-3′) [4] with Illumina adapter sequences. Amplifications were performed in 20 µL reactions with 5× HOTFIRE Pol Blend Master Mix (Solis BioDyne, Tartu, Estonia). Paired-end sequencing of 2 × 300 nucleotides of this pool was performed using a MiSeq Reagent kit v3 on an Illumina MiSeq system (Illumina Inc., San Diego, CA, United States) at the Department of Soil Ecology, Helmholtz Centre for Environmental Research. The ITS rDNA sequences corresponding to the forward and reverse primers were trimmed from the demultiplexed raw reads using cutadapt [5]. Paired-end sequences were quality-trimmed, filtered for chimeras, and merged using the DADA2 package [6] through the pipeline dadasnake [7] Assembled reads fulfilling the following criteria were retained for further analyses: minimum lengths of 70 nt, quality scores at least equal 9 with maximum expected error score of 5 for forward and reverse sequences and no ambiguous nucleotides. Merging was 5 conducted with 2 mismatches allowed and a minimum overlap of 20 nucleotides required for fungal sequences. High-quality reads were clustered into 2,686 amplicon sequence variants (ASVs), after chimera removal. Fungal ASVs were classified against the UNITE v7.2 database [8–10]. All ASVs were classified using the Bayesian classifier as implemented in the mothur classify.seqs command, with a cut-off of 60 [11]. Rare ASVs (singletons or sequences that was observed in only one sample), which potentially represent artificial sequences, were removed. The read counts were rarefied to 71,330 reads. Finally, we obtained 2,683 rarefied fungal ASVs. Rarefaction curves of all the samples indicated the sufficient sampling effort (Fig. S1); thus, we used the observed richness as a measure of fungal diversity associated with PBSA degradation. Ecological functions of fungi were determined for each ASV using FungalTraits [12, 13]. 318 fungal ASVs were assigned to have plant pathogen as primary lifestyle.

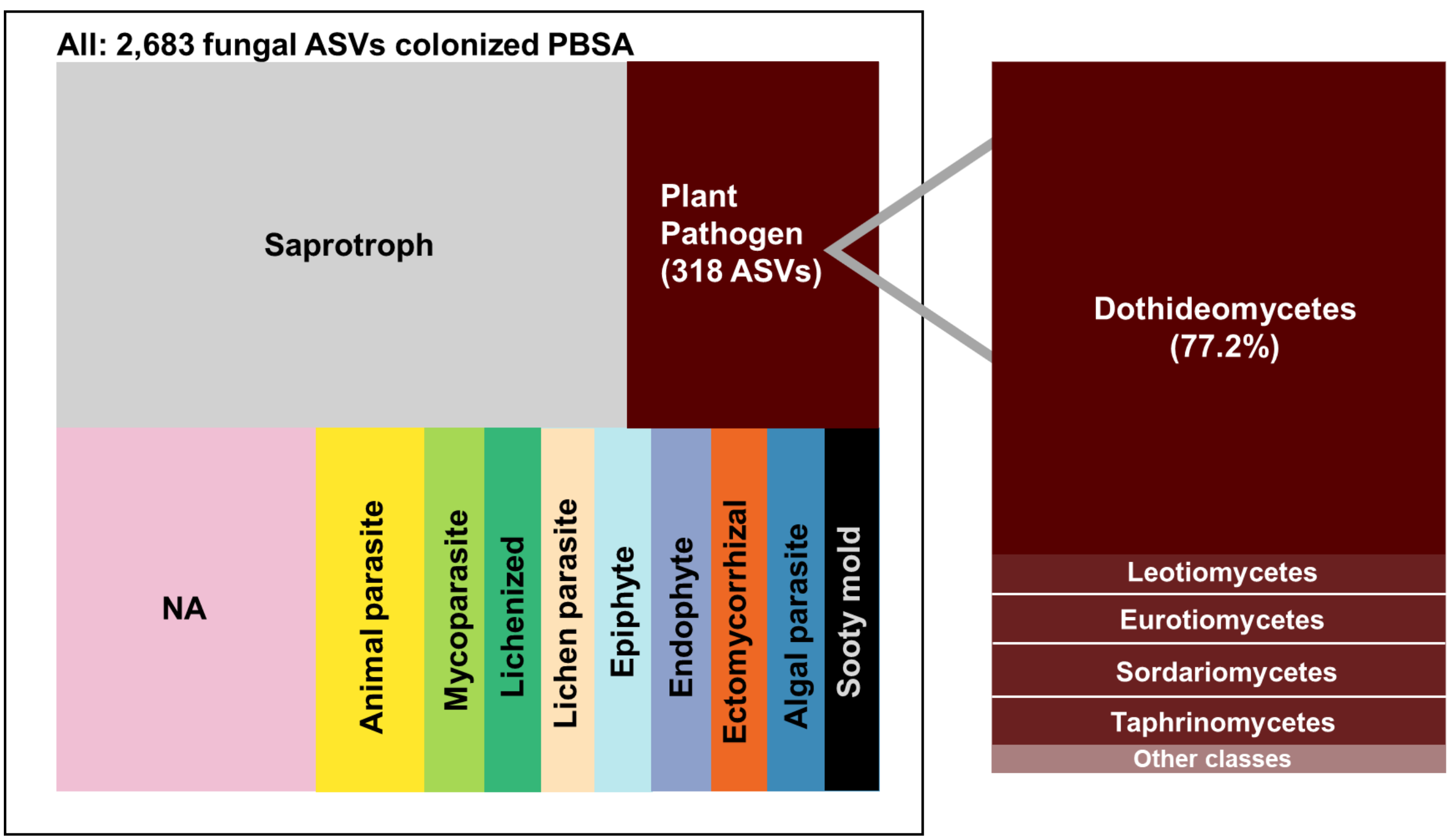

Statistics

In this study, we tested the effects of time, forest type (broadleaved and coniferous tree species) and tree species (the four tree species) on fungal plant pathogen richness and community composition, as well as the link between fungal plant pathogenic community compositions and different environmental factors (including soil pH, soil water content, latitude, longitude, and physicochemical properties of leaf litter layer). Effects of time, and forest type on fungal plant pathogenic ASV richness were assessed through repeated ANOVA analysis using SPSS. Effects of tree species and forest type on fungal plant pathogenic community composition as well as the relationship between fungal plant pathogenic community compositions and different environmental factors (including tree species, soil pH, soil water content, coordinates, and physicochemical properties of litter layer) was analyzed using a goodness-of-fit statistic based on observed relative abundance and the Bray–Curtis distance measure.

Table S1 Information on relative abundance (%) of fungal ASVs detected in poly(butylene succinate-co-adipate) (PBSA) incubated in forest ecosystems. Please see in another excel file.

Table S2 Information on relative abundance (%) of fungal plant pathogen ASVs in class level. Please see in another excel file.

Table S3 Information on relative abundance (%) of fungal plant pathogen ASVs in genus level. Please see in another excel file.

Table S4 Goodness-of-fit statistics (R^2) of environmental and PBSA variables fitted to the nonmetric multidimensional scaling (NMDS) ordination of fungal plant pathogenic community composition based on relative abundance data across all tree species. *P< 0.05, **P< 0.01, and ***P< 0.001.

**Factors**	**All tree types**
Tree factors	
Tree type	0.51***
Tree species	0.53***
PBSA factor	
Timepoint	0.54***
Plot factors	
Soil water content	0.38***
Soil pH	0.32***
Latitude	0.66***
Longitude	0.34***
Leaf litter layer factors	
Leaf litter layer water content	0.52***
Leaf litter layer pH	0.31**
C	0.32***
N	0.02
Ca	0.05
Fe	0.46***
K	0.21**
Mg	0.45***
P	0.22*
C: N ratio	0.04
C: P ratio	0.20**
N: P raio	0.35**

Table S5 Average relative abundance of fungal plant pathogens detected in PBSA samples under 4 tree species. Please see in another excel file.

Figure S1 Rarefaction curves of fungi.

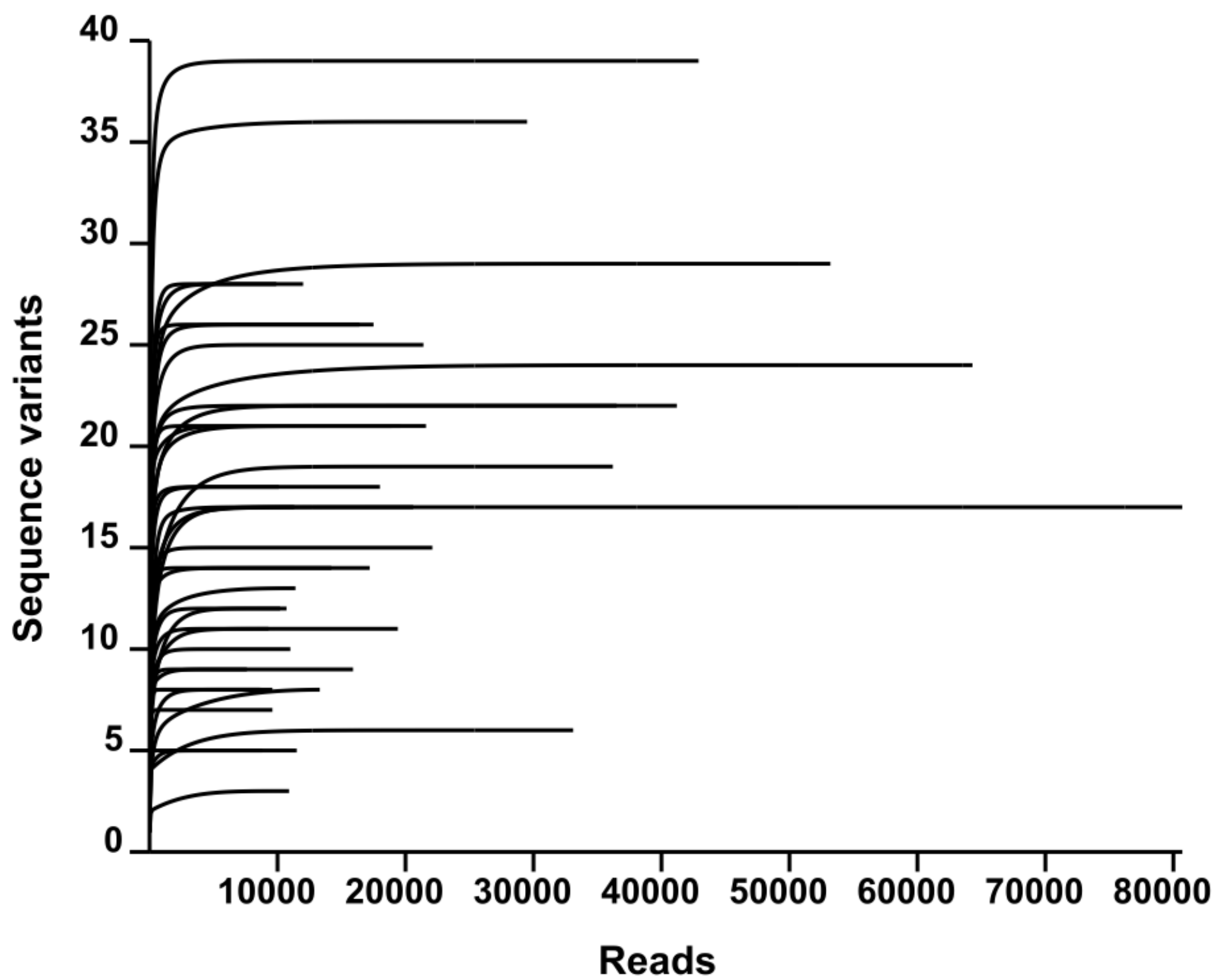

Figure S2 Number of fungal plant pathogenic ASVs identified across different numbers of tree species in a temperate forest ecosystem. The diagram illustrates the distribution of ASVs associated with 1, 2, 3, or 4 tree species.

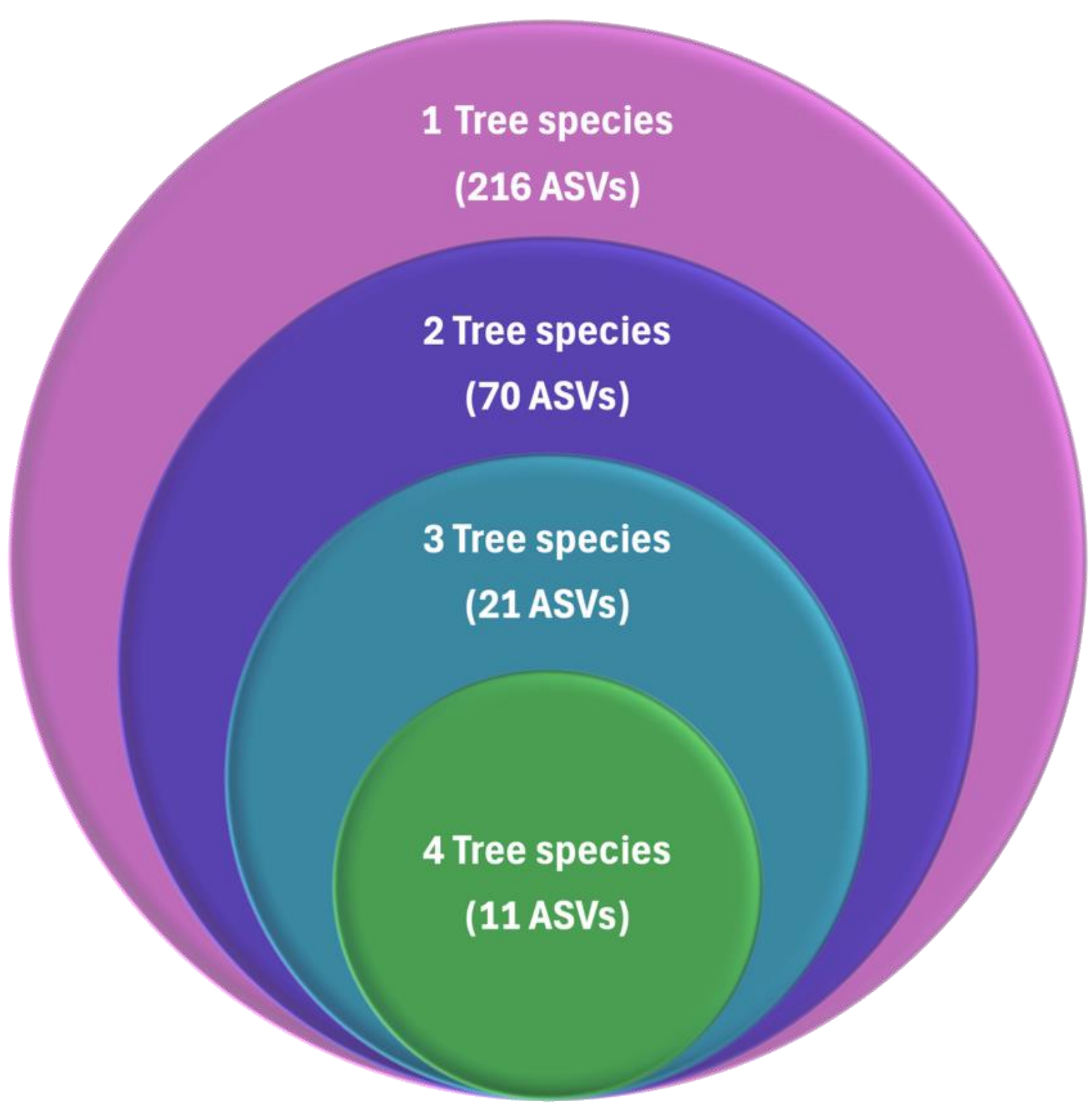

References

1. Purahong W, Wahdan SFM, Heinz D, et al (2021) Back to the Future: decomposability of a biobased and biodegradable plastic in field soil environments and its microbiome under ambient and future climates. Environ Sci Technol 55:12337–12351. https://doi.org/10.1021/acs.est.1c02695
2. Tanunchai B, Juncheed K, Wahdan SFM, et al (2021) Analysis of microbial populations in plastic–soil systems after exposure to high poly(butylene succinate-co-adipate) load using high-resolution molecular technique. Environ Sci Eur 33:105. https://doi.org/10.1186/s12302-021-00528-5
3. Ihrmark K, Bödeker ITM, Cruz-Martinez K, et al (2012) New primers to amplify the fungal ITS2 region - evaluation by 454-sequencing of artificial and natural communities. FEMS Microbiol Ecol 82:666–677. https://doi.org/10.1111/j.1574-6941.2012.01437.x
4. White TJ, Bruns T, Lee S, Taylor J (1990) Amplification and direct sequencing of fungal ribosomal RNA genes for phylogenetics. PCR Protocols 31:315–322. https://doi.org/10.1016/b978-0-12-372180-8.50042-1
5. Martin M (2011) Cutadapt removes adapter sequences from high-throughput sequencing reads. EMBnet Journal, 17:10–12. https://doi.org/10.14806/ej.17.1.200.
6. Callahan BJ, McMurdie PJ, Rosen MJ, et al (2016) DADA2: High-resolution sample inference from Illumina amplicon data. Nat Methods 13:581–583. https://doi.org/10.1038/nmeth.3869
7. Weißbecker C, Schnabel B, Heintz-Buschart A (2021) Dadasnake, a snakemake implementation of DADA2 to process amplicon sequencing data for microbial ecology. Gigascience 9:giaa135. https://doi.org/10.1093/gigascience/giaa135
8. Kõljalg U, Nilsson HR, Schigel D, et al (2020) The taxon hypothesis paradigm—On the unambiguous detection and communication of taxa. Microorganisms 8:1–24. https://doi.org/10.3390/microorganisms8121910
9. Kõljalg U, Nilsson RH, Abarenkov K, et al (2013) Towards a unified paradigm for sequence-based identification of fungi. Mol Ecol 22:5271–5277. https://doi.org/10.1111/mec.12481
10. Nilsson RH, Larsson KH, Taylor AFS, et al (2019) The UNITE database for molecular identification of fungi: Handling dark taxa and parallel taxonomic classifications. Nucleic Acids Res 47:D259–D264. https://doi.org/10.1093/nar/gky1022
11. Wang Q, Garrity GM, Tiedje JM, Cole JR (2007) Naïve Bayesian classifier for rapid assignment of rRNA sequences into the new bacterial taxonomy. Appl Environ Microbiol 73:5261–5267. https://doi.org/10.1128/AEM.00062-07
12. Põlme S, Abarenkov K, Henrik Nilsson R, et al (2020) FungalTraits: a user-friendly traits database of fungi and fungus-like stramenopiles. Fungal Divers 105:1–16. https://doi.org/10.1007/s13225-020-00466-2
13. Tanunchai B, Ji L, Schroeter SA, et al (2023) FungalTraits vs. FUNGuild: Comparison of ecological functional assignments of leaf- and needle-associated fungi across 12 temperate tree species. Microb Ecol 85:411–428. https://doi.org/10.1007/s00248-022-01973-2
